# NBS1 Mutation and prognosis of prostate cancer

**DOI:** 10.1186/1897-4287-10-S4-A29

**Published:** 2012-12-10

**Authors:** Dominika Wokołorczyk, Wojciech Kluźniak, Cezary Cybulski, Jan Lubiński

**Affiliations:** 1 Department of Genetics and Pathomorphology of the Pomeranian Medical University, Szczecin, Poland

## 

Inherited factors contribute to the burden of prostate cancer, however the identification of susceptibility genes for prostate caner has been challenging. To establish the contribution of eight founder alleles in three DNA damage repair genes (BRCA1, CHEK2 and NBS1) to prostate cancer in Poland, and to measure the impact of these variants on survival among patients, 3750 men with prostate cancer and 3956 cancer-free controls were genotyped for 3 founder alleles in BRCA1 (5382insC, 4153delA, C61G), 4 alleles in CHEK2 (1100delC, IVS2+1G>A, del5395, I157T), and 1 allele in NBS1 (657del5). Strong associations were seen for both CHEK2 and NBS1. BRCA1 was not associated with the risk of prostate cancer, NBS1 mutation was associated with poor survival - mortality was significantly worse for carriers of a NBS1 mutation than for non-carriers (HR = 1.85; p = 0.008). We conclude that a founder mutation in NBS1 predisposes to aggressive prostate cancer.

